# Looks Like VT But Isn't - Successful Ablation Of A Left Free Wall Accessory Pathway With Mahaim-like Properties

**Published:** 2009-03-15

**Authors:** Faizel Osman, Peter J Stafford, G Andre Ng

**Affiliations:** 1 University Hospital Coventry, Department of Cardiology, Clifford Bridge Rd, Coventry CV2 2DX; 2 Glenfield Hospital, University Hospital of Leicester, Groby Rd, Leicester, LE3 9QP

**Keywords:** atrio-fascicular accessory pathways, Mahaim-like pathway, Mahaim pathway

## Abstract

It was long believed that Mahaim pathways represented nodo-fascicular or nodo-ventricular connections. However, this misconception was challenged when patients underwent surgical or catheter ablation of the AV node but remained pre-excited. Electrophysiology (EP) studies showed these pathways to be right sided decrementally conducting atrio-fascicular accessory pathways with the atrium forming a part of the antidromic tachycardia circuit. Mahaim pathways are usually reported to occur on the right side. We report a patient who presented with a broad complex tachycardia thought to be ventricular tachycardia; however during EP study this was shown to be an antidromic atrioventricular tachycardia utilising a left free wall pathway that demonstrated 'Mahaim-like' properties and was successfully ablated. The pathway was shown to have long conduction times with no retrograde conduction, had an effective refractory period longer than the AV node and its conduction was only evident during antidromic AVRT. It also had a decremental antegrade property and was responsive to intravenous adenosine. These 'Mahaim-like' features are very unusual and rarely reported on the left side.

## Introduction

 Mahaim fibres are accessory pathways that usually cause an antidromic atrio-ventricular re-entrant tachycardia (AVRT) with a left bundle branch block pattern. We report a case of a patient presenting with a broad complex tachycardia and right bundle branch block pattern that was thought to be ventricular tachycardia on resting 12-lead electrocardiogram (ECG) initially but was found to be due to an antidromic AVRT utilising a left sided accessory pathway with Mahaim-like properties that was successful ablated.

## Case Report

 A 44-year old woman presented with a two hour history of sudden onset palpitation while walking her dog but no syncope. She had no significant past medical history of note but a paternal uncle had died suddenly at the age of 50 years and her father had been diagnosed as having cardiomyopathy and had an implantable cardioverter defibrillator implanted several years previously. She was taking no regular medication. On arrival into hospital she had a blood pressure of 110/60 mmHg and pulse rate of 215 beats per minute; there were no signs of cardiac failure. A resting 12-lead electrocardiogram (ECG) revealed a regular broad complex tachycardia, which was diagnosed as ventricular tachycardia (Figure 1a); this diagnosis was based on previously reported criteria [1]. The patient reverted to sinus rhythm spontaneously and repeat 12-lead ECG revealed no abnormalities (Figure 1). Transthoracic echocardiography and exercise tolerance test were normal. Coronary angiography and cardiac magnetic resonance imaging scan were also normal.

 The patient was referred for electrophysiological assessment and treatment. Following counselling and informed consent, she was admitted for electrophysiology study which was carried out under local anaesthesia and intravenous sedation. Catheters were inserted via femoral venous access with a decapolar catheter (Irvine Biomedical Inc, California, USA) placed in the coronary sinus (CS) and quadripolar catheters (Bard Electrophysiology, MA, USA) placed at the right ventricular apex and His bundle position. Programmed stimulation demonstrated concentric, decremental retrograde and antegrade conduction. A spontaneous tachycardia was observed during the study with the same morphology as the clinical tachycardia (Figure 2a). The tachycardia cycle length was 447 ms and V:A time was 246 ms with earliest atrial activation at His bundle / proximal CS position (Figure 2b); there was no His bundle signal preceding ventricular signal during tachycardia. Earliest local ventricular activation during tachycardia was at the distal CS suggesting either a ventricular tachycardia with 1:1 retrograde atrial activation or an antidromic atrioventricular re-entry tachycardia involving a left free wall accessory pathway. A late atrial premature stimulus delivered from CS 7-8 reset ventricular activation without fusion during tachycardia (Figure 3a) supporting the diagnosis of an antidromic AVRT. Intravenous adenosine (6 mg) terminated the tachycardia with last activation in the atrium (Figure 3b) suggesting adenosine-sensitive property in the antegrade limb of the tachycardia (i.e. the accessory pathway). A trans-septal approach was taken to map the location of the accessory pathway during antidromic tachycardia with an irrigated tip deflectable ablation catheter (D curve Thermocool, Biosense-Webster, USA). The mitral annulus was mapped during tachycardia with earliest ventricular activation identified at the left free wall. Ablation caused termination of tachycardia which was still inducible after ablation cessation. A retrograde approach was then used with successful ablation achieved at a location with good unipolar signal (Figure 4a and4b). There was no recurrence of tachycardia following ablation and tested with programmed stimulation with intravenous isoproterenol infusion. The accessory pathway demonstrated unusual characteristics: it had no retrograde conduction, was slowly conducting with conduction only apparent in the antegrade direction in tachycardia, and had an effective refractory period longer than the AV node. These features are consistent with 'Mahaim-like' properties and have rarely been reported for left sided pathways.

## Discussion

 In 1938, Mahaim described the existence of islands of conducting tissue extending from the AV node into the ventricular myocardium [2]. Since then it has long been believed that Mahaim pathways represented nodo-fascicular or nodo-ventricular connections. This misconception was challenged when patients underwent surgical or catheter ablation of the AV node but remained pre-excited. A series of critical observations made in electrophysiological, surgical, and histopathology laboratories showed these pathways could be ablated surgically, and unlike traditional AV accessory pathways were located away from the AV groove in the majority of cases.

Electrophysiology studies confirmed these pathways to be right sided decrementally conducting atrio-fascicular accessory pathways with the atrium forming a part of the antidromic tachycardia circuit [3]. Several features of these pathways (such as decremental antegrade conduction, minimal or no pre-excitation during sinus rhythm, absence of retrograde conduction over the pathway, responsiveness to adenosine, participation of the right atrium in the antidromic AV re-entrant tachycardia) provide evidence to support the hypothesis that these fibres represent accessory or ectopic AV nodal conduction system consisting of a proximal component similar to the AV node at or above the tricuspid annulus, which connects to a distal component that generate the Mahaim pathway potential [3]. Occurrence of Mahaim automatic rhythm with QRS morphology similar to the fully pre-excited QRS complex, spontaneously or during radiofrequency ablation of the pathway has been reported [4].

 Mahaim pathways are usually reported to occur on the right side. Only sporadic cases of left-sided antegrade decremental pathways have been reported including one left posterior [5], one left postero-septal [6], two left free-wall [7,8], four left nodo-fascicular or nodo-ventricular pathways [9-11] and one on the supero-septal aspect of the mitral annulus [12]. In our patient the pathway was found to be at the left free wall. The pathway was shown to have long conduction times with no retrograde conduction, had an effective refractory period longer than the AV node and its conduction was only evident during antidromic AVRT. It also had a decremental antegrade property and was responsive to intravenous adenosine. These 'Mahaim-like' features are very unusual and have rarely been reported for a left free wall pathway. We did not demonstrate all of the features of a Mahaim pathway (i.e. Mahaim potential and automaticity). The utility of a late atrial premature stimulus, which reset the tachycardia, was important in establishing the correct diagnosis. Patients with Mahaim accessory pathways are commonly believed to be protected against rapid ventricular response during atrial arrhythmias. However, short R-R intervals during atrial fibrillation have been reported in Mahaim patients, indicating that decremental conduction does not fully protect all patients from dangerously high ventricular rates [7].

## Conclusions

We report a left free wall pathway with slow decremental antegrade conduction which was only evident during antidromic AVRT which was initially thought to be ventricular tachycardia. There was no retrograde conduction of the pathway and it was responsive to adenosine; these features have rarely been reported for a left free wall pathway and may represent a variant demonstrating 'Mahaim-like' properties. The utility of a late atrial premature stimulus which reset the tachycardia is highlighted in making the correct diagnosis.

## Figures and Tables

**Figure 1a:**
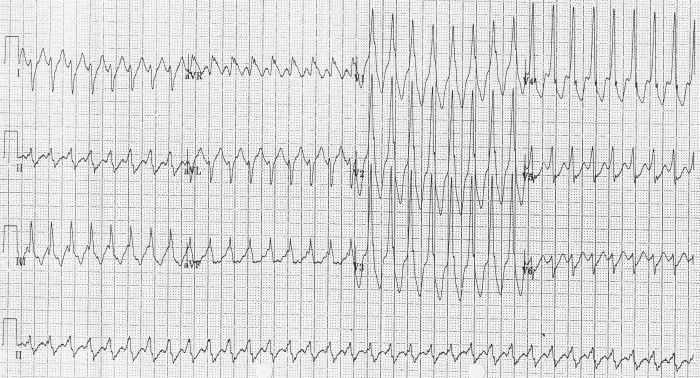
Resting 12-lead ECG showing broad complex tachycardia

**Figure 1b:**
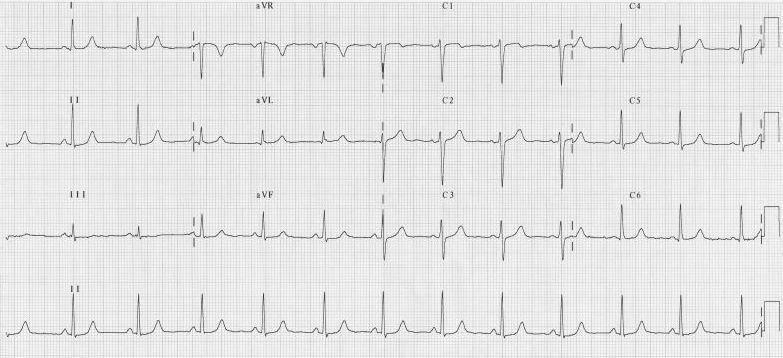
Resting 12-lead ECG after spontaneous reversion to sinus rhythm

**Figure 2a:**
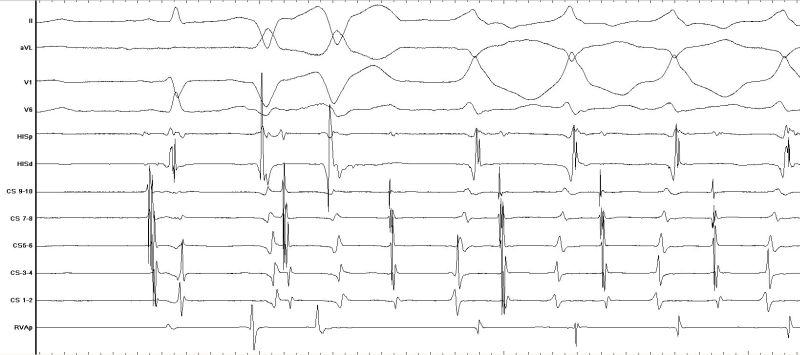
Tachycardia onset during EP study initiated by 2 ventricular ectopics with a left bundle branch block type morphology

**Figure 2b:**
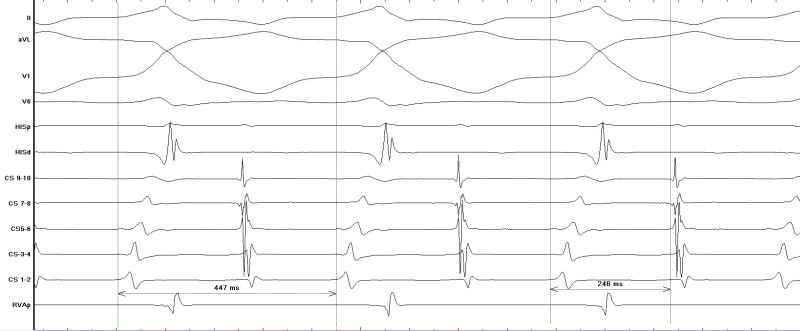
Measurement of cycle length and V:A time during tachycardia

**Figure 3a:**
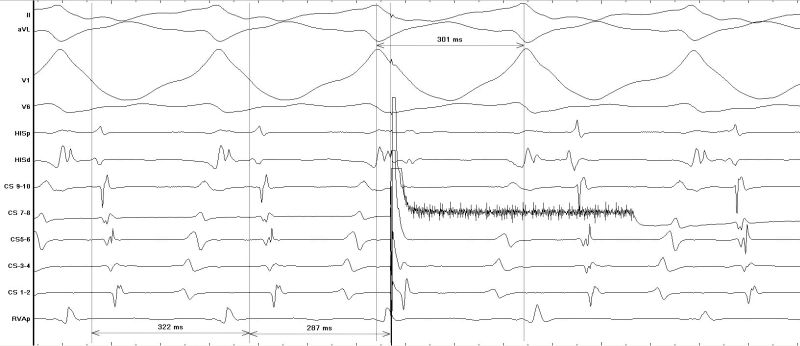
A late atrial premature stimulus delivered from CS 7-8 resets ventricular activation without fusion during tachycardia supporting the diagnosis of an antidromic AVRT

**Figure 3b:**
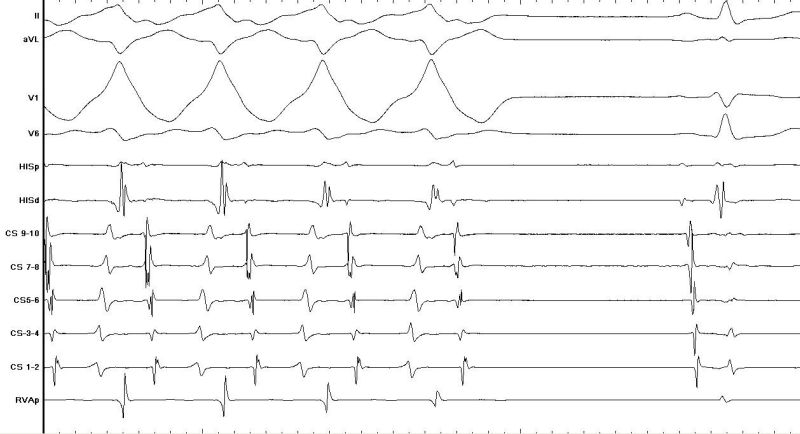
Intravenous adenosine terminates tachycardia with last activation in the atrium showing the adenosine-sensitive property of the accessory pathway

**Figure 4a:**
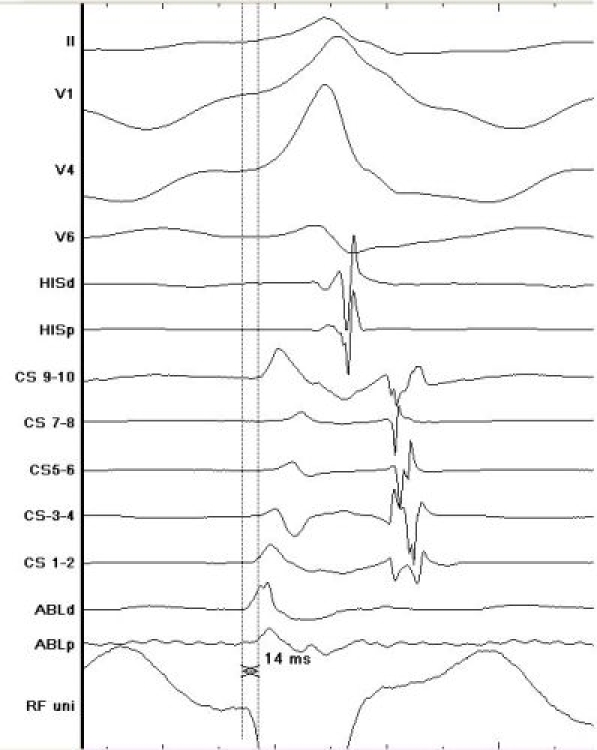
Site of successful ablation demonstrating a good ablation signal that precedes earliest delta wave on surface ECG by 14msec as well as a good unipolar signal

**Figure 4b:**
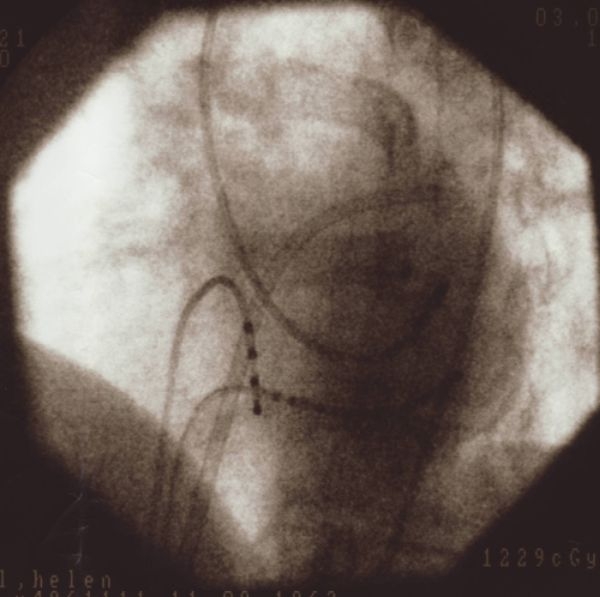
Left anterior oblique projection (40°) showing retrograde catheter placement at site of successful ablation
